# Influence of Mangosteen Peel Extract on Oxidative Stability, Nutritional Values, Physicochemical Properties and Sensory Preference of Soy-Based Burgers

**DOI:** 10.17113/ftb.63.01.25.8629

**Published:** 2025-03

**Authors:** Thy Quynh Bao Nguyen, Nguyen Hoang Khoa Nguyen, Nhu Bich, Linh Tran Khanh Vu, Ngoc Lieu Le

**Affiliations:** 1 Department of Food Technology, International University, Quarter 6, Linh Trung Ward, Thu Duc City, Ho Chi Minh City, Vietnam; 2 Vietnam National University, Ho Chi Minh City, Vietnam; 3 Department of Food Technology, Faculty of Chemical Engineering, Ho Chi Minh City University of Technology, 268 Ly Thuong Kiet, District 10, Ho Chi Minh City, Vietnam; 4 Department of Food Technology, Faculty of Chemical and Food Technology, Ho Chi Minh City University of Technology and Education, Ho Chi Minh City, Vietnam

**Keywords:** shelf life, TBARS, phenols, peroxide, carbonyl, consumer acceptability

## Abstract

**Research background:**

Despite being a substantial and expanding market segment, there remain challenges concerning the shelf life of plant-based meat alternatives when synthetic preservatives are not used. Consequently, it is necessary to investigate the integration of natural extracts into these products to extend their shelf life.

**Experimental approach:**

The total phenolic content, flavonoid content and antioxidant capacity of the powder of dried mangosteen peel extract was characterised. The fresh soy-based burgers were then formulated for six treatments including control (no antioxidant added), 10 mg butylated hydroxytoluene (BHT, a synthetic antioxidant), 10, 7.5, 5 and 2.5 mg dried extract and their proximate composition, physicochemical characteristics, protein and lipid oxidation, texture profile and sensory parameters during 10 days of storage were evaluated.

**Results and conclusions:**

The addition of the extract reduced the moisture content and cooking loss. In addition, the burgers with the extract (5–10 mg/100 g) had remarkably lower values of peroxides, thiobarbituric acid reactive substances and carbonyls, indicating their higher stability against lipid and protein oxidation. These effects of the extract proved to be better than those of BHT. The burgers containing the extract also had improved texture in terms of springiness, chewiness and cohesiveness, resulting in higher texture scores. All treatments were accepted by consumers, with the average score of approx. 7 to 9 points. Therefore, the extract from mangosteen peels could be used as an excellent natural antioxidant substitution for synthetic ones currently used in food preservation.

**Novelty and scientific contribution:**

The study fulfils a need for the growing plant-based meat alternatives with an extended shelf life of a healthier version by using a natural antioxidant extract from mangosteen peels instead of synthetic butylated hydroxytoluene. In addition, the study provides an assessment of product quality during storage and presents findings that could drive innovation in the use of natural preservatives in the food industry.

## INTRODUCTION

It is widely recognised that transitioning from meat-dominant diets to plant-based alternatives is essential to reduce the adverse impact of the food system on the environment, while simultaneously improving human health outcomes and promoting animal welfare. Excessive consumption of meat products has been linked to various health problems such as obesity, type 2 diabetes, cardiovascular disease and certain cancers (*1*). It also contributes to environmental problems such as increased greenhouse gas emissions and the degradation of terrestrial and aquatic biodiversity (*2*), which can lead to climate change (*3*). In contrast, plant-based meat alternatives can reduce overall environmental impact and limit the effect on public health (*4*). With this in mind, more than 4400 plant-based meat alternatives have recently been introduced worldwide to increase the daily consumption of products made from plants (*5*).

Several types of meat substitutes are available on the market such as sausages, burgers, tofu, bacon, steaks and meatballs (*6*). Vegetarian burgers are of interest to consumers as they are ready-to-eat products with ease of preparation (*7*). Thanks to this convenience, burgers have become a familiar fast food in everyday life, which in turn makes the preservation and extension of the shelf life of the product the main goal to consider (*8*). The quality and shelf life of vegan burgers are mainly determined by the oxidation of lipids due to the presence of edible oils in the formulation (*9*), and the products usually have a short shelf life in cold storage without the addition of antioxidants. Therefore, synthetic antioxidants like butylated hydroxyanisole (BHA) or butylated hydroxytoluene (BHT) are introduced, which are responsible for the inhibition of fat oxidation in burger products (*9*). However, these antioxidants raise numerous health concerns due to their potential to cause cancer and carcinogenesis. This has led to an increasing demand for innovative research on organic extracts as alternatives (*10*), such as plant extracts rich in vitamins, carotenoids (carotene, lycopene and astaxanthin), polyphenols and flavonoids.

Mangosteen (*Garcinia mangostana*) is a tropical plant that is widely cultivated in south-east Asian countries (*11*). Mangosteen peels are considered a good source of bioactive compounds such as phenolic acids, xanthones (nonpolar compounds), flavonoids and condensed tannins or pro-anthocyanidins (polar compounds), which have mainly antioxidant and medicinal properties. However, research to date has mostly focused on investigating the components of mangosteen peel extracts for pharmaceutical applications (*12*), while the use of bioactive compounds of this extract in food has not yet been investigated. Therefore, the aim of this study is to examine the efficiency of mangosteen peel extract in preventing lipid and protein oxidation of soy-based burgers and extending their shelf life. The effects of this extract on the nutritional composition, physicochemical properties, texture profile and sensory preferences of the products were also investigated.

## MATERIALS AND METHODS

### Materials and chemicals

Dried mangosteen (*Garcinia mangostana*) peel was purchased from Thanh Binh Herbal Tea Co., Ltd (Ho Chi Minh City, Vietnam). The ingredients used for soy burger formulation were purchased at a local supermarket, except for textured vegetable soy protein and food additives, which were ordered from VINASING Science Development Co., Ltd and Green Cosmetics Store (both Ho Chi Minh City, Vietnam), respectively. Chemicals of the analytical grade were supplied by local distributors, where Folin–Ciocalteu reagent and 2,2-diphenyl-1-picrylhydrazyl (DPPH) were purchased from Sigma-Aldrich, Merck (St. Louis, MO, USA), dinitrophenyl hydrazine (DNPH), guanidine hydrochloride and sodium phosphate were from Acros (Geel, Belgium), trichloroactic acid (TCA), 2-thiobarbituric acid (TBA), potassium sulfate, acetic acid, glacial acetic acid, ethanol, sodium hydroxide and copper(II) sulfate were purchased from Thermo Fisher Scientific (Ward Hill, MA, USA), aluminium chloride, boric acid, ethyl acetate, sodium thiosulfate, starch solution and sodium carbonate were from Guanghua Sci-Tech Co. Ltd. (Guangdong, PR China), ascorbic acid, chloroform, hydrochloric acid, Tashiro’s indicator, potassium iodine, sulfuric acid, hexane and sodium chloride were from Xilong Scientific Co., Ltd., (Guangdong, PR China) and 2,6-di-tert-butyl-4-methylphenol (BHT) was from Quzhou Ebright Chemicals Co., Ltd. (Zhejiang, PR China).

### Extraction of phenols from mangosteen peels

The mangosteen peel extract was prepared following a modified method of Zhou *et al.* (*13*). Initially, 5 g of dried mangosteen peels were ground into a powder and sieved through a 250-μm sieve. The powder was extracted with 75 mL of *V*(ethanol):*V*(distilled water)=70:30 at 60 °C for 15 min. The resulting mixture was centrifuged (Universal 320R; Hettich, Tuttlingen, Germany) at 2377×*g* and 4 °C for 15 min to obtain the filtrate. The filtrate was then further concentrated using a rotary evaporator (Steroglass, Perugia, Italy) at 100 rpm and 70 °C until no more solvent could be removed. The concentrated extract of mangosteen peel was frozen for 24 h and then freeze-dried (Gamma 2-16 LSCplus; Martin Christ, Osterode am Harz, Germany). The dried powder was kept in zipper bags with moisture absorbent pads (5 g per bag; LPS Vina, Ho Chi Minh City, Vietnam) and stored in a freezer until further use.

### Soy burger preparation

Soy-based burgers were processed according to the method of Trujillo-Mayol *et al.* (*14*). Briefly, the formulation consisted of 70 % textured vegetable soy protein (hydrated at a ratio of 1:2 by mass) combined with 30 % of an emulsion comprising 40 % sunflower oil, 30 % egg white, 19.5 % water, 10 % starch and 0.5 % salt. The formulation was divided into six groups. The lyophilised powder of mangosteen peel extract was added to the burger formulation at four different mass fractions (2.5, 5, 7.5 and 10 mg/100 g). The fifth group included BHT (10 mg/100 g) as a positive control and the last group did not contain any antioxidant source (negative control). BHT was added to the burger formulation at 10 mg, which is in agreement with the guidelines by the Vietnamese Ministry of Health for the management of food additives (*15*).

The burger patties, weighing (50±2) g each, were formed using a burger press kitchen mould with thickness of 1 cm and 5 cm in diameter. They were then wrapped in oxygen-permeable PVC film and kept refrigerated at 4 °C. To determine cooking loss and sensory attributes, the burgers were fried in a pan. Six burger patties were evenly distributed along the edges of a preheated pan to maintain uniform cooking conditions. The burgers were fried for 6 min, flipped once at the 3-minute mark and continued frying until the internal temperature reached 75 °C.

### Determination of total phenolic content, total flavonoid content and antioxidant activity of the dried mangosteen peel extract

To determine the total phenolic content (TPC) and total flavonoid content (TFC), 1 g of the powder of peel extract was dissolved with 100 mL ethanol *φ*=70 %. TPC was determined using the Folin-Ciocalteu method (*16*) and expressed in mg of gallic acid equivalents (GAE) per g of dried extract. In brief, 0.5 mL of extract was mixed with 0.5 mL of 10 % (*m*/*V*) Folin-Ciocalteu solution, vortexed (VM-10; Daihan, Seoul, South Korea) at approx. 1000 rpm for 10 s and left for 5 min. After that, 0.5 mL of 7.5 % (*m*/*V*) sodium carbonate solution was added to the mixture, followed by 2.25 mL of distilled water. The mixture was kept at room temperature in the dark for 30 min before measuring the absorbance at 765 nm using a spectrophotometer (V-770 UV/Vis/NIR; Jasco, Tokyo, Japan).

On the other hand, TFC was estimated using the aluminium chloride colourimetric method (*17*) and expressed in mg of rutin equivalents (RE) per g of dried extract. In brief, 1 mL of extract was mixed with 0.3 mL of 5 % (*m*/*V*) NaNO_2_. The mixture was allowed to stand for 5 min and then 0.3 mL of 10 % (*m*/*V*) AlCl_3_ was added. After incubation for 5 min, 2 mL of 1 M NaOH and 1.4 mL of distilled water were added and the absorbance was then measured at 510 nm.

The antioxidant activity was determined using the DPPH radical scavenging capacity (*18*). The peel extract powder and BHT were dissolved in methanol *φ*=80 % to prepare different concentrations. The volume of 1 mL of each concentration was mixed with 3 mL of 0.1 mM DPPH in methanol and vortexed (VM-10; Daihan) at approx. 1000 rpm for 10 s and left for 40 min. The absorbance was then measured at 517 nm. The half maximal inhibitory concentration (IC_50_) was calculated from the linear regression of the curve plotting between the percentage of inhibition and the antioxidant concentrations.

### Proximate composition, cooking loss, mass loss and pH of soy burgers

Proximate composition (moisture, ash, protein, lipids, and carbohydrate content) of soy burgers was measured according to the standard methods (*19*). Cooking loss was measured as described by Moghtadaei *et al.* (*20*) by weighing the soy burgers before and after frying. Mass loss of soy burgers during refrigerated storage was calculated on day 1, day 5 and day 10 as described by Ganhão *et al.* (*21*). For pH determination, a sample of 10 g was homogenised with 40 mL of distilled water for 1 min. The homogenate was filtered before measuring the pH (*22*).

### Lipid and protein oxidation in soy burgers

Peroxide value (PV, mmol per kg of sample) and thiobarbituric acid reactive substances (TBARS, expressed in nmol of malondialdehyde (MDA) per g of sample) were measured as lipid oxidation parameters, while protein carbonyl content was used as an indicator of protein oxidation. The PV was measured following a standard method (*23*), where the fat was extracted with the solvent *V*(acetic acid):*V*(chloroform)=2:1 and PV was determined by titration. For the determination of TBARS, the analysis was performed according to the method of Sobral *et al.* (*24*) and the results were expressed as nmol MDA per g of sample. Firstly, 5 g of sample were homogenised with 20 mL of 7.5 % (*m*/*m*) TCA and 10 mL BHT (4.5 % *m*/*V* in ethanol) for 5 min. The supernatant (2 mL), which was obtained by centrifuging (Universal 320R; Hettich) at 2377×*g* for 10 min, was mixed with 2 mL of 40 mM TBA in glacial acetic acid. The mixture was heated in a water bath at 90 °C for 45 min. After cooling at room temperature for 10 min, the absorbance was measured at 532 nm.

Carbonyl content was measured based on the traditional DNPH spectrophotometric assay described by Özer and Secen (*25*) and expressed as nmol of carbonyl per mg of protein. Briefly, 5 g of sample were homogenised with 50 mL of 20 mM sodium phosphate buffer (pH=6.5) containing 0.6 M NaCl. Two equal aliquots of 25 mL were then mixed with 5 mL of ice-cold 10 % (*m*/*V*) TCA to precipitate the protein before centrifugation (Universal 320R; Hettich) at 2377×*g* and 4 °C for 5 min. After decanting the supernatant, one pellet was treated with 1 mL of 2 M HCl and the other with 1 mL of 0.2 % (*m*/*V*) DNPH in 2 M HCl. Both samples were incubated for 1 h at room temperature and simultaneously vortexed (VM-10; Daihan) at approx. 1000 rpm for 10 s every 15 min. After the reaction, the samples were precipitated with 5 mL of 10 % (*m*/*V*) TCA and then centrifuged (Universal 320R; Hettich) at 2377×*g* and 4 °C for 5 min. The collected pellets were washed three times with 2 mL of the solvent *V*(ethanol):*V*(ethyl acetate)=1:1. The pellets were then dissolved with 5 mL of 20 mM sodium phosphate buffer containing 6 M guanidine hydrochloride salt (pH=6.5). Centrifugation (Universal 320R; Hettich) was carried out at 2377×*g* and 4 °C for 2 min to remove insoluble fragments. The absorbance of the final solution was measured at 280 and 370 nm.

### Texture profile and sensory tests of soy burgers

The texture profile of soy-based burgers was analysed using a CT3 texture analyzer from Brookfield Engineering Laboratories, Inc. (Middleborough, MA, USA). Samples measuring 2.5 cm×2.5 cm×1 cm from each formulation were compressed in two cycles at room temperature, moving at a constant speed of 1 mm/s to 75 % of their original height. The texture profile analysis included the following parameters: hardness, cohesiveness, springiness and chewiness.

The hedonic test described by Kazemi *et al.* (*26*) was used for sensory evaluation. Thirty panellists were recruited, including students and staff (22 females and 8 males) from the International University, Ho Chi Minh City, Vietnam, aged 18–50. The panellists evaluated the burgers in individual booths under white fluorescent light. They were asked to rate their preferences regarding the appearance, texture, odour, taste, colour and overall acceptability of the burgers using a 9-point hedonic scale, where a score of 9 indicated extreme liking, 5 indicated neutrality, and 1 indicated extreme disliking. They were offered water to rinse their mouths between each sample. The samples were coded and served randomly.

### Statistical analysis

All measurements were carried out in triplicate. One-way analysis of variance was performed using Minitab software (*27*) with Fisher’s *post-hoc* test or independent *t*-test to compare means at a significant level of 95 %.

## RESULTS AND DISCUSSION

### Bioactive properties of mangosteen peel extract

Table 1 shows the data for the bioactive compounds of the dried extract. Total phenolic mass fraction, expressed as gallic acid equivalents (GAE), was 311.3 mg/g dried extract and total flavonoid mass fraction, expressed as rutin equivalents (RE), was 176.1 mg/g dried extract. These values were comparable to those reported for the phenolic extract from cloves (TPC of 456 mg/g and TFC, as catechin equivalents, of 100 mg/g) used to preserve beef burgers (*28*), or to those of pomegranate peel extract (TPC of 392 mg/g and TFC, as quercetin equivalents, of 104 mg/g) used to preserve minced beef (*29*) and higher than those of green leafy vegetable extracts (TPC of 4.53-27.19 mg/g) for the preservation of meat products (*30*). The high TPC and TFC values of the dried extract resulted in high antioxidant capacity, with an IC_50_ of 31.7 mg/mL. In comparison, the IC_50_ of BHT, a commonly used synthetic antioxidant, was 41.7 mg/mL. It can be concluded that the antioxidant capacity of the dried mangosteen peel extract was 1.3-fold higher than that of BHT. Since the recommended mass fraction of BHT to be added to burgers is 10 mg/100 g, the mass fraction of the dried extract to be analysed varied from 2.5 to 10 mg/100 g.

**Table 1:** Antioxidant property of the mangosteen peel extract

Antioxidant property	Quantity
TFC as *w*(RE)/(mg/g)	176.1±80.5
TPC as *w*(GAE)/(mg/g)	311.3±10.5
Antioxidant activity (IC_50_)/(mg/mL)	31.7±2.0
Antioxidant activity of BHT (IC_50_)/(mg/mL)	41.7±2.2

TFC=total flavonoid content, RE=rutin equivalent, TPC=total phenolic content, GAE=gallic acid equivalent, BHT=butylated hydroxytoluene

### Proximate composition and cooking loss of soy-based burgers

Table 2 summarises the proximate composition (on dry mass basis) of the soy-based burgers. The addition of different mass fractions of the extract and BHT altered the moisture content of the burgers, but no significant differences (p>0.05) were observed compared to that of the negative control sample, except for a slight decrease (p<0.05) recorded for the burger with 10 mg dried extract. A slight decrease in moisture content was also reported for other foods with added plant extracts (*31*, *32*). As a result of moisture variation, the fat content of some samples was significantly different. However, the variations were small and amounted to less than 0.7 %. In general, the proximate composition of all samples was approx. 37–38 % protein, 12 % fat, 5 % ash and 44–45 % carbohydrate. This result was similar to the study by Savadkoohi *et al.* (*33*) for a type of plant-based meat prepared from soy protein isolate and egg albumin as ingredients, where the protein, fat, ash and carbohydrate contents were approx. 35, 14, 5 and 44 %, respectively.

**Table 2:** Proximate composition and cooking loss of soy burgers

	*w*(dried mangosteen extract)/(mg/100 g)								
*w*(parameter)/%	C-	C+		10		7.5		5	2.5
Protein	(38.2±0.5)^a^	(37.3±0.6)^a^	(37.5±0.1)^a^		(37.4±0.2)^a^		(38.2±0.1)^a^		(38.1±0.5)^a^	
Fat	(12.1±0.2)^a^	(12.1±0.1)^a^	(11.7±0.2)^ab^		(11.5±0.3)^b^		(11.7±0.2)^ab^		(11.4±0.2)^b^	
Ash	(5.2±0.11)^a^	(5.0±0.2)^a^	(5.1±0.1)^a^		(5.0±0.1)^a^		(5.2±0.1)^a^		(5.1±0.1)^a^	
Carbohydrate	(44.5±0.7)^a^	(45.6±0.7)^a^	(45.8±0.3)^a^		(46.0±0.5)^a^		(45.0±0.1)^a^		(45.5±0.4)^a^	
Moisture	(62.7±0.2)^ab^	(62.0±0.5)^bc^	(61.9±0.4)^c^		(62.0±0.1)^bc^		(62.8±0.1)^a^		(62.7±0.1)^ab^	
Cooking loss	(6.7±0.11)^a^	(6.7±0.1)^a^	(5.7±0.1)^d^		(6.2±0.1)^c^		(6.4±0.1)^b^		(6.4±0.1)^b^	

C-=no antioxidant, C+=10 mg butylated hydroxytoluene. Mean values in the same row with different letters in superscript are significantly different (p<0.05)

Cooking loss refers to the loss of liquid (moisture and fat) and other juices from soy burger patties before and after frying. This parameter was related to different factors such as cooking time, type and amount of ingredients in the formulation. Table 2 shows that the addition of the extract resulted in lower (p<0.05) cooking loss, especially for the samples with 10 mg dried extract, which had the lowest percentage of cooking loss (5.7 %), while the control and BHT had the highest values, *i.e.* 6.7 %. The changes in the cooking loss were mainly due to protein denaturation, which resulted in lower water- and fat-holding capacities and thus increased loss of water and fat (*34*). It was also found that the percentage of cooking loss decreased with an increase in the amount of the extract. The reduced cooking loss could be due to the improved emulsion stability and binding capacity of the extract components, such as polyphenols, to retain water and fat within the matrix. Improved water- and fat-holding capacity and reduced cooking loss have also been reported previously for foods to which phenol-rich plant extracts have been added (*28*, *35*).

### Mass loss and pH value of stored soy burgers

Mass loss is considered as the reduction in the mass of products during refrigerated storage and the collected data are shown in Table 3. Due to moisture evaporation, the mass loss of all samples increased with the prolonged storage time. The addition of dried extract at 10 mg/100 g was able to reduce the mass loss compared to the control (p<0.05), which was consistent with the results in Table 2.

**Table 3:** Physicochemical parameters (mass loss and pH) of stored soy burgers

		*w*(dried mangosteen extract)/(mg/100 g)									
Parameter	*t*/day	C^-^	C^+^	10		7.5		5		2.5	
*w*(mass loss)/%	1	(0.13±0.02)^aC^	(0.14±0.00)^aC^		(0.19±0.04)^aC^		(0.19±0.07)^aC^		(0.21±0.09)^aC^		(0.18±0.03)^aC^
	5	(0.35±0.03)^bB^	(0.51±0.02)^aB^		(0.50±0.06)^aB^		(0.44±0.01)^aB^		(0.47±0.02)^aB^		(0.48±0.03)^aB^
	10	(1.43±0.04)^aA^	(1.40±0.00)^abA^		(1.32±0.05)^cA^		(1.36±0.01)^abcA^		(1.40±0.02)^abA^		(1.33±0.03)^bcA^
pH	1	(6.95±0.07)^bB^	(6.70±0.02)^cB^		(7.17±0.03)^aB^		(7.16±0.01)^aB^		(7.13±0.01)^aB^		(7.11±0.01)^aB^
	5	(7.08±0.04)^bAB^	(6.76±0.02)^cB^		(7.20±0.01)^aAB^		(7.19±0.01)^aAB^		(7.18±0.02)^aAB^		(7.08±0.02)^bB^
	10	(7.14±0.02)^bA^	(6.90±0.03)^cA^		(7.25±0.02)^aA^		(7.22±0.02)^aA^		(7.23±0.01)^aA^		(7.20±0.01)^abA^

C-=no antioxidant, C+=10 mg butylated hydroxytoluene. Mean values in the same row with different lower-case letters in superscript are significantly different (p<0.05). Mean values in the same column with different capital letters in superscript are significantly different (p<0.05)

The pH of the soy burgers was also an important factor that contributed to the quality of the final products and the data are shown in Table 3. The initial pH of the control burger sample was 6.95. This value was considered higher than that of ordinary meat-based products (*e.g.* beef), which is around 5.5. This is the intrinsic property of several plant-based meat products due to the slight alkalinity of their ingredients. In this study, the main ingredients of soy-based burgers were textured vegetable soy protein (pH=7.42–7.43) (*36*) and egg white (pH=7.6–7.9) (*37*), resulting in their high pH value. The neutral or higher pH values were also reported previously for other meat analogues (*38*, *39*). Meanwhile, the burger samples with added dried extract showed significantly (p<0.05) higher pH values (7.11–7.17), while the lowest value was observed with the addition of BHT (6.70). The dried extract was well mixed with the emulsion containing the egg white before it was combined with the hydrated soy protein. There may be an interaction between the components of the extract (*e.g.* phenolic compounds) and those of egg white (*40*) that could destabilise its natural buffering system and cause a pH shift to a slightly higher value (*41*). During storage, the pH slightly increased in all samples. This increase could be due to the volatile bases from the possible decomposition of nitrogen containing compounds (*42*). However, the pH changes in this study were small, less than 0.2 over 10 days of storage. The pH increase during storage in protein-rich food has also been observed in previous studies (*43*).

### Lipid and protein oxidation in stored soy burgers

Table 4 shows the changes in peroxide value (PV), an indicator of primary lipid oxidation, in soy-based burger samples with or without antioxidants during storage. The PV increased with increasing storage time from day 1 to day 10. The negative control soy burger samples showed significantly (p<0.05) higher PVs than the samples with added BHT or dried extract at any time during storage. In addition, the PV of the treated samples on day 10 (2.7–3.1 mmol/kg) were still lower than that of untreated samples on day 1 (3.3 mmol/kg). The increase in PV observed during storage was due to the formation of hydroperoxides during the initial stage of lipid oxidation. It was found that the sample with 10 mg dried extract had significantly lower PV (p<0.05) than the positive control sample containing BHT, while the lower extract mass fractions (2.5–7.5 mg) resulted in equivalent effects to BHT. Therefore, it can be assumed that the extract can effectively slow down fat oxidation at an appropriate amount. This could be due to the high content of phenolic compounds such as flavonoids, xanthones, anthocyanins, *etc.* in the extract.

**Table 4:** Peroxide value (PV), thiobarbituric acid reactive substances (TBARS) and carbonyl content of soy-based burgers during storage

		*w*(dried mangosteen extract)/(mg/100 g)					
Parameter	*t*/day	C-	C+	10	7.5	5	2.5
*b*(PV) /(mmol/kg)	1	(3.3±0.2)^aB^	(2.1±0.2)^bB^	(1.8±0.3)^bB^	(2.1±0.3)^bB^	(2.4±0.3)^bA^	(2.6±0.5)^abA^
	5	(3.8±0.3)^aAB^	(2.82±0.07)^bA^	(2.14±0.07)^dAB^	(2.44±0.04)^cdAB^	(2.7±0.1)^bcA^	(2.9±0.2)^bA^
	10	(4.3±0.2)^aA^	(3.0±0.1)^bA^	(2.68±0.07)^cA^	(2.8±0.2)^bcA^	(3.0±0.1)^bcA^	(3.07±0.07)^bA^
TBARS as *b*(MDA) /(nmol /g)	1	(0.42±0.02)^aC^	(0.32±0.00)^cdC^	(0.29±0.02)^dC^	(0.33±0.01)^bcC^	(0.35±0.00)^bcC^	(0.35±0.00)^bC^
	5	(0.75±0.04)^aB^	(0.58±0.01)^bB^	(0.44±0.01)^eB^	(0.46±0.01)^deB^	(0.51±0.03)^cdB^	(0.54±0.01)^bcB^
	10	(1.64±0.01)^aA^	(1.27±0.01)^bA^	(1.17±0.02)^dA^	(1.18±0.00)^dA^	(1.23±0.00)^cA^	(1.26±0.01)^bcA^
*b*(carbonyl) /(nmol/mg)	1	(0.38±0.04)^aC^	(0.37±0.01)^abC^	(0.30±0.02)^cC^	(0.31±0.03)^bcB^	(0.34±0.02)^abcC^	(0.37±0.01)^aC^
	5	(0.72±0.01)^aB^	(0.52±0.02)^bB^	(0.44±0.01)^dB^	(0.48±0.01)^cA^	(0.49±0.00)^bcB^	(0.50±0.01)^bcB^
	10	(0.81±0.01)^aA^	(0.72±0.02)^bA^	(0.53±0.00)^cdA^	(0.54±0.02)^dA^	(0.57±0.01)^cA^	(0.58±0.02)^cA^

C-=no antioxidant, C+=10 mg butylated hydroxytoluene. Mean values in the same row with different lower-case letters in superscript are significantly different (p<0.05). Mean values in the same column with different capital letters in superscript are significantly different (p<0.05)

The peroxides formed in the first stage of lipid oxidation could be further degraded to secondary products, which were measured by TBARS values. Table 4 also shows the changes in TBARS values of soy-based burger during storage. For all samples, TBARS values were observed to increase (p<0.05) during storage, indicating that secondary oxidation products were accumulated more and more. The change pattern in TBARS was consistent with that of PV. Negative control samples had significantly (p<0.05) higher TBARS values than the burger samples treated with BHT and dried extract at any storage period. After 10 days of storage, the samples containing 5 mg/100 g extract had lower TBARS values than those treated with BHT. Considering both primary and secondary lipid oxidation stages (PV and TBARS), 5 mg of the extract was able to prevent lipid oxidation better than 10 mg of BHT.

In addition to lipid oxidation, protein oxidation in the burger samples during storage was also measured by means of the carbonyl concentration (*44*). Table 4 also shows changes in their concentration during storage. After one day of storage, the carbonyl concentration was not remarkably different among samples, with a variation of less than 0.09 nmol/mg. During storage, the carbonyl concentration in the samples with and without the addition of BHT increased more rapidly than in all samples with the added extract. On day 10, the values of the negative and positive control samples of 0.81 and 0.72 nmol/mg, respectively, were significantly higher (p<0.05) than those of the samples treated with the extract (0.53–0.58 nmol/mg). The increase in carbonyl concentration observed in all samples during storage could be attributed to both physical and chemical interactions between proteins and various reactive species such as free radicals (*e.g.* ROS) and non-radical compounds (*e.g.* H_2_O_2_ and ROOH) generated during lipid oxidation (*45*). In addition, the better efficiency of the extract in retarding protein oxidation could be due to the abundant presence of phenolic compounds, which were able to either bind with proteins, form complexes with them, or inhibit lipid oxidation (*46*). These phenolic compounds could include α-mangostin, β-mangostin, γ-mangostin, chlorogenic acid, vanillic acid, protocatechuic acid, ferulic acid, *etc*., which have been previously identified in mangosteen peel extracts (*47*).

### Texture profile of soy burgers

Among the texture profile attributes, hardness evaluates the force required to induce deformation, chewiness evaluates the force necessary to masticate solid food to a swallowable consistency, cohesiveness evaluates the internal resilience of the food structure and springiness evaluates the elasticity (*32*). Table 5 shows the texture profiles of the soy-based burgers compared between day 1 and day 10. On day 1, none of the samples had significantly different hardness (p>0.05), which was in the range of 9.2–11.5 N/mm^2^. However, the addition of BHT or the extract seemed to increase the springiness, chewiness and cohesiveness of soy burgers, except for the addition of 10 mg. These increases could be attributed to the interaction between BHT or phenolic compounds in the extract and protein molecules, resulting in a stronger fibrous network (*48*). However, an excess of polyphenols could cause the network to become excessively rigid, leading to a decrease in these properties, as reported by Ma and Ryu (*32*). Comparing the formulations on day 1 and day 10, the hardness of the negative control sample increased, while it decreased slightly or remained unchanged in the other samples. The increase in the hardness of the negative control during storage could be related to the oxidative damage of the proteins, which leads to the formation of protein carbonyls and crosslinking of the proteins (*49*). Meanwhile, the springiness, chewiness and cohesiveness of all samples remained unchanged or increased during storage, except for the 10 mg addition. This confirmed again that the excessive addition of the extract can significantly alter the structural network of the burger and its rearrangement behaviour during storage.

**Table 5:** Texture profile of soy-based burgers

		*w*(dried mangosteen extract)/(mg/100 g)					
Parameter	*t*/day	C^-^	C^+^	10	7.5	5	2.5
Hardness/N	1	(11.5±1.0)^aB^	(10.6±1.5)^aA^	(9.2±3.0)^aA^	(10.5±1.2)^aA^	(10.8±0.7)^aA^	(10.4±1.4)^aA^
	10	(14.3±0.4)^aA^	(9.1±2.2)^bA^	(9.0±0.6)^bA^	(9.1±0.0)^bB^	(7.7±1.0)^cB^	(8.3±1.1)^bcB^
Springiness/mm	1	(2.4±0.2)^cB^	(2.7±0.1)^abB^	(2.6±0.1)^abcB^	(2.7±0.0)^abA^	(2.8±0.1)^aB^	(2.6±0.1)^bcB^
	10	(2.8±0.1)^bA^	(2.9±0.0)^abA^	(3.0±0.0)^aA^	(2.8±0.0)^bA^	(2.9±0.1)^abA^	(2.8±0.1)^bA^
Chewiness/(N·mm)	1	(18.7±4.1)^aB^	(22.7±4.4)^abA^	(17.2±5.8)^bA^	(22.6±2.0)^abA^	(24.0±1.0)^aA^	(19.7±2.5)^abA^
	10	(33.8±3.1)^aA^	(23.8±4.7)^abA^	(10.6±0.2)^cB^	(29.4±7.8)^abA^	(20.2±1.8)^bcA^	(20.8±3.3)^bcA^
Cohesiveness/%	1	(0.7±0.1)^bB^	(0.79±0.05)^aB^	(0.72±0.04)^abA^	(0.79±0.02)^abB^	(0.80±0.03)^aB^	(0.75±0.02)^abB^
	10	(0.84±0.03)^bA^	(0.90±0.03)^abA^	(0.40±0.03)^cB^	(0.85±0.00)^abA^	(0.90±0.01)^aA^	(0.89±0.01)^aA^

C-=no antioxidant, C+=10 mg butylated hydroxytoluene. Mean values in the same row with different lower-case letters in superscript are significantly different (p<0.05). Mean values in the same column with different capital letters in superscript are significantly different (p<0.05)

### Sensory evaluation

Sensory preference of consumers for soy-based burgers was evaluated based on their scores for sensory attributes including appearance, colour, odour, texture, taste and overall acceptability. The results in Table 6 show that no statistical differences were found for five of the six sensory attributes, with the exception of texture, where the average score for each attribute was between 6 and 7. This suggests that the addition of BHT or the extract would not significantly change the sensory quality of the burgers. Regarding texture, the addition of the dried extract appeared to increase the consumers’ scoring of the resulting burgers compared to the negative and positive BHT control samples, especially of the burger with 10 mg dried extract, whose score was significantly better (p<0.05). The higher preference could be due to the water-holding capacity of both textured soy proteins and phenols in the extract, which improved springiness, chewiness and cohesiveness, as explained in Table 5. In conclusion, all burgers with or without added preservatives had the overall acceptability score of approx. 7 from maximum 9 points, which means that they have a high chance of being accepted by consumers.

**Table 6:** Sensory evaluation including appearance, colour, odour, taste, texture and overall acceptability of soy-based burgers on a 9-point scale

	*w*(dried mangosteen extract)/(mg/100 g)					
Attribute	C-	C+	10	7.5	5	2.5
Appearance	(6.7±1.3)^a^	(6.5±1.6)^a^	(6.3±1.8)^a^	(6.30±1.7)^a^	(6.1±1.5)^a^	(6.2±1.7)^a^
Colour	(6.7±1.1)^a^	(6.6±1.0)^a^	(6.0±1.5)^a^	(6.3±1.6)^a^	(6.0±1.6)^a^	(6.6±1.5)^a^
Odour	(6.8±1.5)^a^	(6.4±1.8)^a^	(6.5±1.5)^a^	(6.6±2.0)^a^	(6.6±2.0)^a^	(6.6±1.7)^a^
Texture	(6.3±1.6)^c^	(6.5±1.5)^bc^	(7.5±1.0)^a^	(7.4±1.0)^ab^	(6.7±1.7)^ab^	(6.7±1.5)^ab^
Taste	(6.9±1.5)^a^	(6.7±1.7)^a^	(6.6±1.8)^a^	(6.9±1.6)^a^	(6.9±1.6)^a^	(6.8±1.5)^a^
Overall acceptability	(7.2±1.1)^a^	(6.9±1.4)^a^	(7.1±1.1)^a^	(7.2±1.5)^a^	(6.7±1.6)^a^	(7.0±1.4)^a^

C-=no antioxidant, C+=10 mg butylated hydroxytoluene. Values in the same row with different letters in superscript are significantly different (p<0.05)

## CONCLUSIONS

The results of this study consistently confirm that dried mangosteen peel extract, which is high in phenolic compounds, is a feasible and effective natural antioxidant. The addition of different mass fractions of the extract of 2.5–10 mg/100 g was evaluated as a safe substitute for artificial antioxidants in soy-based burger formulations to effectively prevent protein and lipid deterioration during ten days of chilled storage. In particular, the highest mass fraction of the extract in soy burgers (*i.e.* 10 mg/100 g) was found to reduce the formation of oxidation products more efficiently than the use of BHT. The control soy-based burger had a high pH close to neutral (6.95), while the addition of the extract caused a slight increase in pH to 7.11–7.17. In terms of sensory preference, the presence of the extract was able to improve the score for texture while maintaining the overall acceptability of the burgers. Consequently, the positive results of this natural antioxidant compared to those of BHT indicate its potential application in the food industry.
